# The Association between Y Chromosome Microdeletion and Recurrent Pregnancy Loss

**Published:** 2012-06-30

**Authors:** S Ghorbian, K Saliminejad, M R Sadeghi, Gh R Javadi, K Kamali, N Amirjannati, F Bahreini, H Edalatkhah, H R Khorram Khorshid

**Affiliations:** 1 Department of Biology, Science and Research Branch, Islamic Azad University, Tehran, Iran; 2 Reproductive Biotechnology Research Center, Avicenna Research Institute, ACECR, Tehran, Iran; 3 Monoclonal Antibody Research Center, Avicenna Research Institute, ACECR, Tehran, Iran; 4 Genetic Research Center, University of Social Welfare and Rehabilitation Science, Tehran, Iran

**Keywords:** Infertility, Recurrent pregnancy loss, Miscarriages, Y chromosome microdeletion, AZF regions

## Abstract

**Background:**

To date, the role of male factor contributing in evaluation of spontaneous recurrent pregnancy loss (RPL) has been less investigated and there is discrepancy in the role of Y chromosome microdeltions in RPL. Therefore, the current study was designed to examine whether Y chromosome microdeletions were associated with RPL in an Iranian population.

**Methods:**

One hundred men from couples, experiencing three or more RPLs, and one hundred normal men from couples with at least one child and no history of miscarriages as control group were included. Genomic DNA was extracted from peripheral blood and tested for Y chromosome microdeletions in AZFa, AZFb and AZFc regions using two multiplex PCR.

**Results:**

None of the men in the case and control groups had any microdeletions in the AZFa, AZFb and AZFc regions.

**Conclusion:**

It seems that Y chromosome microdeletion is not associated with recurrent pregnancy loss, therefore performing this test in Iranian couples with RPL is not recommended.

## Introduction

Recurrent pregnancy loss (RPL) is usually defined as three or more consecutive pregnancy losses, which affects 0.5-3% of all couples.[1][2] RPL is a multifactorial condition with several etiologic factors including genetic abnormalities of the parents, anatomical, endocrinologic, hematologic and immunologic abnormalities, infections, nutritional and environmental factors.[2][3] The causes of pregnancy loss in about half of the women with RPL even after an extensive investigations remain unexplained.[4]

To date, the clinical investigation of couples with RPL has mainly focused on the female partner.[2] The male factor contributing in evaluation of RPL has been less investigated and largely limited to karyotype analysis. There are evidences that male factors can potentially affect fertilization, embryo development, viability and placental proliferation as well as differentiation of trophoblast cells. Paternally expressed genes modulate the proliferation and invasiveness of trophoblast cells and later placental proliferation.[5][6][7][8]

Approximately 1-4% of couples with recurrent miscarriage have cytogenetic abnormalities.[2] Mutations such as small deletions, substitutions, duplications, translocations or point mutations could not be detected by cytogentic analysis. These smaller genetic abnormalities may be account for a lot of miscarriages with unknown causes. Association of Y chromosome microdeletions and male infertility were hypothesized by Tiepolo and Zuffardi in the middle of 70’s.[9] Y chromosome microdeletions are found in 10-15% of men with idiopathic azoospermia and severe oligozoospermia.[10]

According to the recent studies, there is a potential association between Y chromosome microdeletions and RPL.[11][12] The association between Y chromosome microdeletions and RPL are poorly studied. So, the current study was designed to find whether Y chrosomome microdeletions in men with couples experiencing RPL were associated with RPL in an Iranian population.

## Materials and Methods

This is a case-control genetics association study. One hundred men from couples with history of three or more consecutive miscarriages, who referred to Avicenna Infertility Clinic, Tehran, Iran, during 2009-2010, were included. One hundred healthy men from couples with at least one live birth and no miscarriages were considered as control group. Our study was approved by Avicenna Research Institute's Ethics and Human Rights Committee and an informed consent was obtained from each man in the case and control group. In the case group, all men and their spouses were analyzed for the karyotype and those with cytogenetic abnormalities were excluded. All clinical information of couples including history of all previous miscarriage, age and clinical work-up to determine cause of miscarriage was obtained from Avicenna Infertility Clinic. All women with RPL examined with hysterosalpingography and ultrasonography for detection of anatomical abnormalities of the genital tract. Hematological disorders such as, anti-thrombin III activity, proteins C and S activity, activated protein C resistance, factor V Leiden mutation, protrombin mutation, immunological risk factors such as natural killer cells activity (NK Cells), lupus anticoagulant, anticardiolipin antibodies, antinuclear antibodies (ANAs) and for endocrinological disorders, hormone analysis including, follicle-stimulating hormone (FSH), luteinizing hormone (LH), free testosterone, prolactin, thyroid stimulating hormone (TSH) were checked in all women with at least 3 miscarriages.

Genomic DNA was extracted from 3 ml peripheral blood samples using salting-out method.[13] The screening for Y chromosome microdeletions in the AZFa (sY84 and sY86), AZFb (sY127 and sY134) and AZFc (sY254 and sY255) were performed using two multiplex PCR according to the EAA/EMQN guideline with a few modifications.[14] In addition, we tested the two markers sY150 and sY152 (AZFc) which used in the study by Dewan et al.,[11]

**Fig. 1:**
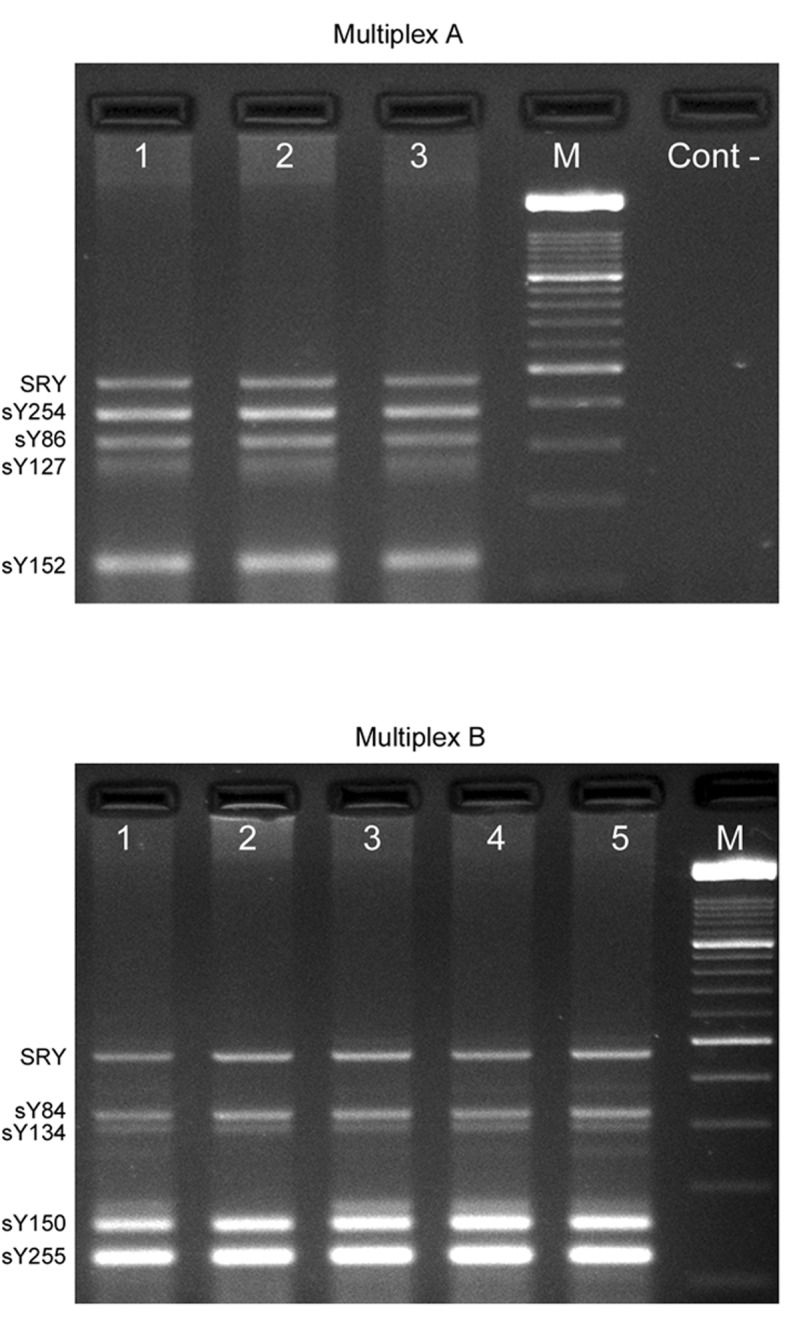
The result of the multiplex A and B. PCR fragments were separated on 3% agarose gel. Lanes 1-2-3, men with couples experiencing RPL; Lanes 4-5, fertile control men; M, Molecular weight marker (100 bp ladder); Cont -, Negative control (Water). SRY (472 bp), sY84 (324 bp), sY86 (326 bp), sY127 (274 bp), sY134 (301 bp), sY254 (380 bp), sY255 (123 bp), sY150 (158 bp), sY152 (125 bp).

Multiplex a reaction contains sY86, sY127, sY254 and sY152 while multiplex B reaction contains sY84, sY134, Y255 and sY150. The SRY gene was used as internal control in both reactions. The sequences of all primer pairs and the size of the PCR products were shown in Table 1. The PCR was carried out in a total volume of 25 µl containing 200 ng of genomic DNA, 1xPCR buffer, 4 mM MgCl_2_, 1U Taq DNA polymerase (Roche; Germany), 10 nmol each NTPs, and 10 pmol of each primers. Amplification conditions were started with an initial denaturation at 95°C for 10 minutes, followed by 40 cycles of denaturation at 94°C for 30 seconds, annealing at 62°C for 45 seconds, and extension at 72°C for 90 seconds, ended by a last extension at 72°C for 10 minutes and cooling to 4°C. The PCR products were separated on 3% Agarose gel, stained with ethidium bromide and visualized under UV light. Genomic DNA from fertile men and water used as positive and negative control, respectively (Figure 1).

**Table 1:** Sequence of the primer pairs used to screen Y microdeletions.

**STS**	**Region**	**Size (bp)**	**Primer sequence**
sY84****	AZF	324****	5’-AGAAGGGTCTGAAAGCAGGT-3’ 5’-GCCTACTACCTGGAGGCTT-3’****
sY86		326	5’-GTGACACACAGACTATGCTTC-3’ 5’-ACACACAGAGGGACAACCCT-3’
sY127	AZF	274	5’-GGCTCACAAACGAAAAGAAA-3’ 5’-CTGCAGGCAGTAATAAGGGA-3’
sY134		301	5’-GTCTGCCTCACCATAAAACG-3’ 5’-ACCACTGCCAAAACTTTCAA-3’
sY254	AZF	380	5’-GGGTGTTACCAGAAGGCAAA-3’ 5’-GAACCGTATCTACCAAGCAGC-3’
sY255		123	5’-GTTACAGGATTCGGCGTGAT-3’ 5’-CTCGTCATGTGCAGCCAC-3’
sY150		158	5’-GGGAGAGTCACATCACTTGG-3’ 5’-TTGAATTATCTGCCTGAGTGC-3’
sY152		125	5’-AAGACAGTCTGCCATGTTTCA-3’ 5’-ACAGGAGGGTACTTAGCAGT-3’
SRY	Yp	472	5’-GAATATTCCCGCTCTCCGGA-3’ 5’-GCTGGTGCTCCATTCTTGAG-3’

Our data were presented as mean and standard deviation. To compare the means between study groups, student's t-test for independent samples and Mann-Whitney U test were used when the data distributions were normal and not normal, respectively. SPSS software (Version 11.5, Chicago, IL, USA) was used for statistical analysis and p values <0.05 were considered statistically significant.

## Results

The clinical characteristics of cases and controls were shown in Table 2 and  3. The mean age of men in the case group was 40 years (SD=4.9) and the mean age of women was 35.5 years (SD=5.0).The mean number of previous abortions was 3.8 (SD=1.2). Y chromosome microdeletion analysis revealed no microdeletion in AZFa, AZFb and AZFc regions in the case and control group.

**Table 2:** FSH, LH and testosterone levels in men with couples experiencing RPL and control group.

**Hormones**	**RPLa(SD)** **N=20**	**Controlb(SD)** **N=20**	**P valuec**
FSH (mlu/ml)	3.65 (2.79)	9.25 (3.04)	0.068
LH (mlu/ml)	5.30 (4.09)	4.4 (1.41)	1
Testosterone (mlu/ml)	12.54 (7.63)	3.15 (1.20)	0.068

^a^ Men with couples experiencing three or more consecutive miscarriages

^b^ Men of fertile couples with at least one live birth and no miscarriages

^c^ By a Mann Whitney U test, p value<0.05

**Table 3:** Sperm concentration and semen volume in men with couples experiencing RPL and control group.

****	**RPL^a^(SD) ** **No.=100**	**Controlb(SD)** **No.=100 **	***P* valuec**
Sperm concentration (10^6^ mill/ml)	45.57 (57.67)	37.97 (25.93)	0.341
Semen volume (ml)	3.16 (1.36)	3.29 (1.46)	0.581

^a^ Men with couples experiencing three or more consecutive miscarriages

^b^ Men of fertile couples with at least one live birth and no miscarriages

^c^ By a student t test, P value<0.05

## Discussion

Recurrent pregnancy loss, is estimated to affect 0.5-3.0% of all couples.[1][2] The cause of about half of all pregnancy losses is often unknown.[7] The clinical investigation of couples with RPL largely has focused on the female partner and male factors potentially contributing to the RPL have been less investigated.[2][15]

Three AZF regions on the long arm of Y chromosome are essential for normal spermatogenesis.[16] AZF deletions have a negative impact on the sperm quality and abnormal spermatozoa that may be associated with RPL.[17][18][19] Based on previous studies, the complete deletion of the AZFb and AZFc may have a direct effect on early prophase and decrease the rate of normal pairing in pachytene stage of spermatocytes. Pairing failure increases chromosomal abnormalities and could be related with recurrent miscarriages.[20][21]

Recent studies showed that Y chromosome microdeletion in AZF region may be a possible etiologic factor of RPL.[11][12] According to the study by Dewan et al. (2006), 82% of RPL couples (14/17) had at least one microdeletion and 65% had three or more microdeletions in AZF regions in the long arm of human Y chromosome.[11]

They used 4 sequence tagged sites: sY67, sY129, sY150 and sY152.[11] The STS marker sY67 used by Dewan et al. is in fact located on the short arm of the Y chromosome and is not inside the AZF regions on the long arm of Y chromosome. They reported that they found patients with deletions of both sY67 and an additional marker. This result indicates that these patients would have multiple deleted regions of the Y chromosome, which is highly unlikely in a normal male.[11][22][23] Karaer et al. (2008) reported that 7/43 and 2/43 patients had microdeletions in the sY129 and sY153, respectively.[12] They used 4 STS: sY129 for AZFb region and sY150, sY152 and sY153 for the AZFd region, but these are not true microdeletions, because individual STS absence should be considered as polymorphisms or methodological mistakes.[14][22]

The aim of the present study was to determine the association between Y chromosome microdeletions and RPL in Iranian population. All 100 patients and 100 control men were checked for AZF microdeletion using 6 STS recommended by EAA/EMQN guideline (2004) and also the two markers sY150 and sY152 which used by Dewan et al.,[11] Y chromosome microdeletions were found neither in the male partners of women experiencing RPL nor in the control men, and therefore our findings does not support the results of studies by Dewan et al. (2006) and Karaer et al. (2008).[11][12]

Despite of the previous study, recently published article by Kaare et al. (2008) with 46 Finish couples and analyzing 32 STS, including EAA/EMQN recommendations and STS used by Dewan et al. (2006) showed absence of Y chromosome microdeletion in male partners.[11] In another recently published article by Wettasinghe et al. (2010), 76 male partners of couples where the female partner had experienced three or more RPL and 120 normal men were analyzed. None of the men had any microdeletions in the AZFa, AZFb, AZFc regions or partial deletions in the AZFc region.[23]

There are important differences between the prevalence of Y chromosome microdeletion and RPL in previous studies. For understanding causes of discrepancies, we must consider that for AZF microdeletions screening, there is validated guideline endorsed by the EAA/EMQN which could detect up to 95% of all reported AZF microdeletions.[14] However, these were not used in the studies carried out by Dewan et al. (2006) and Karaer et al. (2008).[11][12] As this study was performed using DNA extracted from peripheral blood, the possibility remains that abnormalities leading to miscarriage have arisen in the spermatozoa through de novo mutations during spermatogenesis.[24]

Finally, it seems that Y chromosome microdeletions are not associate with RPL and more research is needed, therefore performing this test in Iranian couples with RPL is not recommended.
